# Utility of Cerebral Microvascular Imaging in Infants Undergoing ECMO

**DOI:** 10.3390/children9121827

**Published:** 2022-11-26

**Authors:** Luis Octavio Tierradentro-Garcia, Joseph A. Stern, Rebecca Dennis, Misun Hwang

**Affiliations:** 1 Department of Pediatric Radiology, Children’s Hospital of Philadelphia, Philadelphia, PA 19104, USA; 2 Department of Radiology, Perelman School of Medicine, University of Pennsylvania, Philadelphia, PA 19104, USA

**Keywords:** ECMO, microvascular imaging, cerebral blood flow, brain ultrasound, pediatric neuroradiology

## Abstract

Purpose: Infants who require extracorporeal membrane oxygenation (ECMO) therapy have an increased risk of neurological complications and mortality. Microvascular imaging (MVI) is an advanced Doppler technique that allows high-resolution visualization of microvasculature in the brain. We describe the feasibility and utility of MVI for the evaluation of cerebral microvascular perfusion in patients undergoing ECMO. Methods: We retrospectively analyzed brain MVI scans of neonates undergoing ECMO. Two pediatric radiologists qualitatively assessed MVI scans to determine the presence or absence of tortuosity, symmetry, heterogeneity, engorgement, and hypoperfusion of the basal ganglia–thalamus (BGT) region, as well as the presence or absence of white matter vascular engorgement and increased peri-gyral flow in the cortex. We tested the association between the presence of the aforementioned brain MVI features and clinical outcomes. Results: We included 30 patients, 14 of which were male (46.7%). The time of ECMO duration was 11.8 ± 6.9 days. The most prevalent microvascular finding in BGT was lenticulostriate vessel tortuosity (26/30, 86.7%), and the most common microvascular finding in the cortex was increased peri-gyral flow (10/24, 41.7%). Cortical white matter vascular engorgement was significantly associated with the presence of any poor outcome as defined by death, seizure, and/or cerebrovascular events on magnetic resonance imaging (*p* = 0.03). Conclusion: MVI is a feasible modality to evaluate cerebral perfusion in infants undergoing ECMO. Additionally, evidence of white matter vascular engorgement after ECMO cannulation could serve as a predictor of poor outcomes in this population.

## 1. Introduction

Extracorporeal membrane oxygenation (ECMO) is a rescue therapy for severe, refractory cardiorespiratory conditions and has been associated with a significant increase in morbidity and mortality in the young pediatric population [1]. Neurological injury and complications are major causes of death and long-term disability in neonatal ECMO, with an incidence of 10–52% in neonates and children [2,3,4], and can be associated with different factors, including pre-ECMO injury type and severity, prolonged systemic heparinization, duration of ECMO, and microthrombi from the ECMO circuit [3,4,5,6]. Despite recent technical advances in ECMO, the prevalence of brain injury has not changed, and prognostic information which guides management remains limited [4].

Neurological injury in the setting of ECMO is complex and multifactorial [7]. For instance, direct damage can occur during carotid artery and internal jugular vein cannulation, or after, as a thrombotic event [8]. Anticoagulation, most often unfractionated heparin, is typically used to prevent hypercoagulability and clot formation in the ECMO circuit, but its unbalanced use can lead to hemorrhagic events [9]. Additionally, inflammatory reactions and consumptive coagulopathy after ECMO cannulation can cause altered hemodynamics and ultimately brain injury [7].

In infants, transfontanellar brain grayscale ultrasound (US) is the preferred method for real-time screening of cerebral injury and is often used daily to rule out large intracranial bleeding and help guide the course of ECMO treatment [4,10]. However, this modality is less successful in identifying smaller and non-hemorrhagic lesions [4,5]. Also, there has been a demonstrated discrepancy between the detection of abnormalities with brain US when compared with computed tomography (CT) and magnetic resonance imaging (MRI), raising concerns about the sensitivity of brain US [11,12]. CT is only used for emergency evaluations after an abnormal brain US when findings may impact immediate survival or candidacy of ECMO [13]. MRI is the gold standard for detecting neurological injury in the neonatal population and is especially useful in ECMO survivors to detect hemorrhagic and ischemic lesions in gray and white matter. Prior studies with MRI indicate that white matter is the frontal and temporoparietal lobes is more commonly affected [13,14]. An important disadvantage of MRI is that ECMO equipment is incompatible with MRI and can only be performed after ECMO decannulation when patients often remain critically ill after injury potentially occurred and developed.

Microvascular imaging (MVI) is an innovative Doppler technique that allows the high-resolution visualization of microvasculature by separating low-velocity flow signals from motion artifacts using advanced clutter suppression [15]. MVI of the neonatal brain has been demonstrated to be a useful modality for visualizing cerebral microvasculature [15,16,17]. In this regard, MVI may serve as a noninvasive tool to evaluate the functional status of hemodynamics and flow patterns during ECMO. For example, Hwang and colleagues described altered morphology in the lenticulostriate vessels of a patient under ECMO showing decreased flow and mildly tortuous striatal vessels when compared to a normal patient [15]. However, there is a paucity of literature on the specific vascular features that could be depicted during ECMO and their implications for clinical outcomes. Our primary objective is to describe the feasibility of MVI to evaluate cerebral microvascular perfusion patterns in patients undergoing ECMO; our secondary objective is to determine whether there are associations between brain MVI features and clinical outcomes.

## 2. Materials and Methods

### 2.1. Study Design and Population

This retrospective, cross-sectional, single-center study was conducted in a tertiary pediatric hospital. We used our institutional search engine, Illuminate InSight (Overland Park, KA, USA), to identify infants (age < 1 year) that underwent ECMO between November 2020 and September 2021 at the Children’s Hospital of Philadelphia. We included patients who had at least one MVI exam while on ECMO. Patients were excluded if the quality of images was suboptimal for interpretation (i.e., the field of view did not include the regions of interest). We evaluated the first MVI exam available after ECMO cannulation. The study was conducted under Institutional Review Board approval. Documentation of informed consent was waived. The Strengthening the Reporting of Observational Studies in Epidemiology (STROBE) guidelines were used to prepare this manuscript [18].

### 2.2. Clinical Information and Outcomes

We extracted demographic and clinical information from our electronic chart system (Epic Systems Corporation, Verona, WI, USA). We collected data using REDCap 10.9.0 (Vanderbilt University, Nashville, TN, USA) [19]. Clinical data included dates when ECMO started and ended, ECMO indication, number of days during ECMO, death during ECMO, presence of congenital cardiac disease, and outcomes within a year after ECMO cannulation. Outcomes within a year included death, cerebrovascular events (ischemic and hemorrhagic) as evident on MRI, or seizures. We also extracted data from the ultrasound reports to document any lesions or abnormalities that were evident in grayscale US.

### 2.3. Image Acquisition and Interpretation

MVI exams were acquired using an ultrasound machine with integrated MVI technology, namely LOGIQ™ E10 (General Electric, Milwaukee, WI, USA) (Boston, MA, USA). A single linear probe (L2-9) was used in all exams for image acquisition. All captured images available were used for image interpretation, including still images and cine clips in the sagittal and coronal planes. Two pediatric radiologists (M.H. and R.D.), blinded to patient demographics and clinical information, individually assessed the scans. We defined two regions of interest for the evaluation of microvasculature. Firstly, lenticulostriate microvessels in the basal ganglia–thalamus region (BGT) were qualitatively evaluated for the presence or absence of vessel morphological tortuosity, perfusion intensity (i.e., hypoperfusion vs. normal or hyperperfusion), perfusion symmetry, engorgement (defined as larger caliber vessels containing a greater flow signal than expected), and heterogeneity (defined as irregular vascular pattern rather than normal branching pattern of vessels). Secondly, cortical microvessels were qualitatively evaluated for the presence or absence of white matter vascular engorgement and increased or normal peri-gyral flow. Discrepant cases were evaluated by both pediatric radiologists one month apart from the initial blind evaluation, and final responses were determined in consensus.

### 2.4. Data Analysis

Statistical analyses were conducted using IBM SPSS Statistics for Windows (v. 23; IBM Corporation, Armonk, NY, USA). We report qualitative data as percentages; we tested normality for quantitative data using the Shapiro–Wilk test; quantitative data are presented as median and interquartile range (IQR) except for days on ECMO (mean ± SD). We tested the association between the presence of features as described above in MVI, and clinical outcomes; we used the chi-squared or independent t-test depending on the categories of variables; a *p*-value < 0.05 was considered statistically significant. Finally, we calculated inter-rater reliability (IRR) for sonographic findings between the two reviewers using Cohen’s coefficient Kappa; the level of agreement was considered as none (0–0.2), minimal (0.21–0.39), weak (0.40–0.59), moderate (0.60–0.79), strong (0.80–0.90), and almost perfect (>0.90) [20].

## 3. Results

We included 30 patients, 14 of which were male (46.7%). Age at ECMO start was 2 (IQR 1-10) days, age at first MVI was 10 (IQR 5-14) days, and age at termination of ECMO was 19 (IQR 13-25) days. The duration between initiation of ECMO and the first MVI exam was 4 (IQR 2-6) days. The duration of ECMO was 11.8 ± 6.9 days. Nineteen patients had more than one MVI exam for a total of 70 exams; 18/30 (60%) patients had available cine clips for image interpretation. Half of the patients (*n* = 15) had a history of congenital cardiac disease. Indications for ECMO were respiratory failure with or without pulmonary hypertension (*n* = 18, 60%), cardiac/ventricular failure (*n* = 6, 20%), and failure to wean from cardiovascular bypass after major cardiac surgery (*n* = 6, 20%). Three patients (10%) passed away during ECMO, and a total of seven (23.3%) died within one year from ECMO cannulation. For other outcomes assessed with MRI, seven patients (23.3%) had cerebrovascular events, three (10%) presented with seizures without evidence of acute lesion on MRI, and 13 (43.3%) did not have neurological complications within one year from ECMO cannulation.

**Figure 1:**
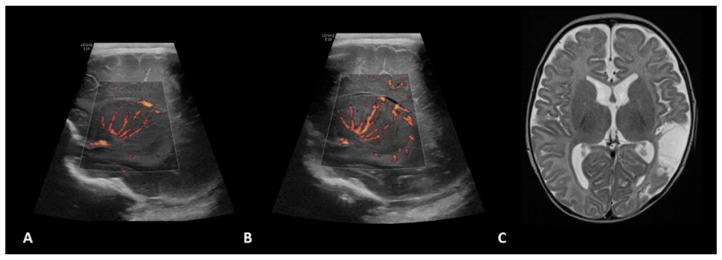
A 1.5-month-old girl with a history of transposition of the great arteries (TGA) underwent ECMO due to failure to wean from cardiovascular bypass after major cardiac surgery. Brain MVI was performed after three days from cannulation; grayscale US (not shown) demonstrated a small focus of increased echogenicity within the left caudate. Right (**A**) and left (**B**) parasagittal brain MVI showed mild bilateral tortuosity of the lenticulostriate vessels. The patient remained on ECMO for a total of four days and presented with seizures two days after decannulation, secondary to a cardioembolic stroke in the territory of the posterior left middle cerebral artery, as shown in axial T2-weighted MRI (**C**) obtained two months later.

**Figure 2:**
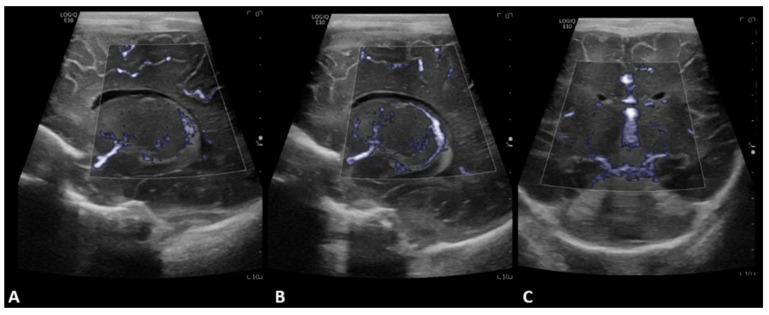
A 2-day-old boy with no history of congenital cardiac disease underwent ECMO due to acute hypoxemic respiratory failure. Brain MVI was performed after three days from cannulation; grayscale US was normal. Left (**A**) and right (**B**) parasagittal, and coronal (**C**) brain MVI demonstrated symmetric decreased blood flow in the basal ganglia.

**Figure 3:**
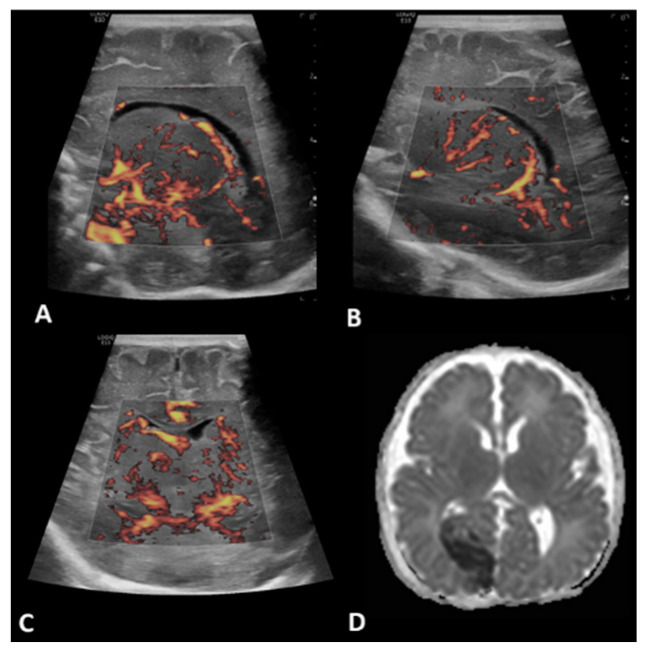
A 2-day-old boy with no history of congenital cardiac disease underwent ECMO due to severe pulmonary hypertension and respiratory failure. Brain MVI was performed after six days from cannulation; grayscale US was normal. Brain MVI demonstrated asymmetric and heterogeneous blood flow in the deep gray nuclei as seen in left (**A**) and right (**B**) parasagittal views, as well as in the coronal view (**C**). The patient remained on ECMO for a total of seven days. MRI obtained two days after ECMO decannulation showed diffusion restriction in the right medial occipital lobe consistent with acute stroke in the expected distribution of branches of the right posterior cerebral artery (**D**).

**Figure 4:**
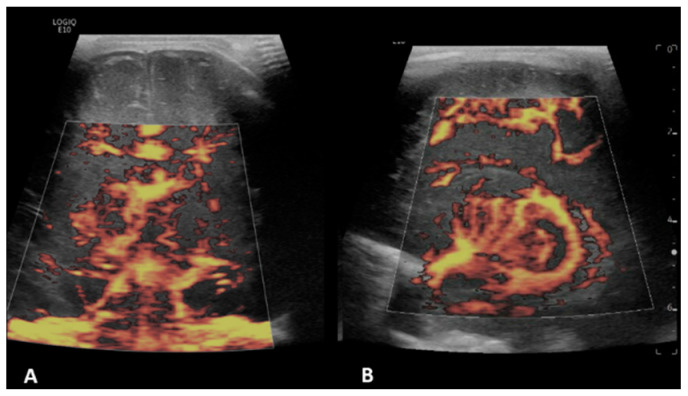
A 11-day-old boy with a history transposition of the great arteries (TGA) underwent ECMO due to failure to wean from cardiovascular bypass after major cardiac surgery. Brain MVI was performed after two days from cannulation; grayscale US (not shown) demonstrated diffuse/patchy hyperechogenicity in the white matter. Coronal (**A**) and right parasagittal (**B**) brain MVI of the basal ganglia and thalamic region demonstrated engorgement of the lenticulostriate microvessels, as well as heterogeneity and asymmetry.

**Figure 5:**
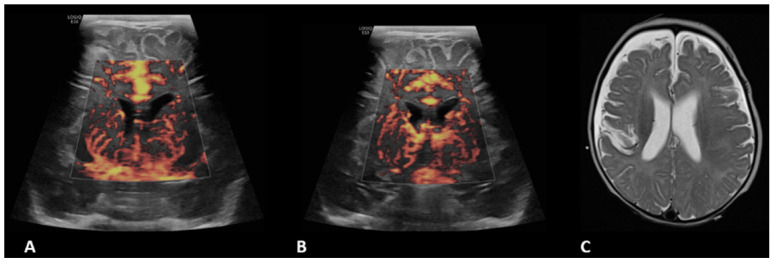
A 4-month-old boy with a history of complete common atrioventricular canal underwent ECMO due to failure to wean from cardiovascular bypass after major cardiac surgery. Brain MVI was performed after five days from cannulation; grayscale US (not shown) demonstrated extra-axial collections. Coronal brain MVI (**A**,**B**) demonstrated marked cortical white matter vascular engorgement and heterogeneity. MRI obtained three months after ECMO decannulation showed a moderate, late subacute to chronic infarct in the right parietotemporal cortex (**C**).

**Figure 6:**
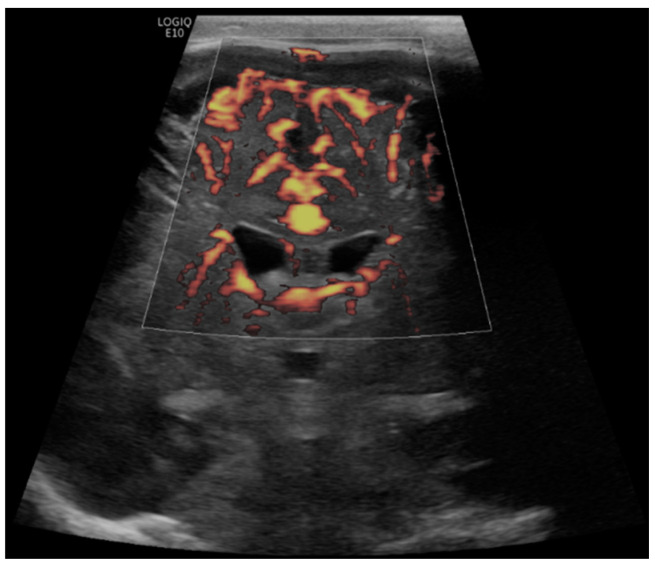
A 6-month-old boy with a history of hypoplastic left heart syndrome underwent ECMO due to failure to wean from cardiovascular bypass after major cardiac surgery. Brain MVI was performed after nine days from cannulation; grayscale US (not shown) demonstrated a hyperechogenic focus within the right posterior temporal lobe/occipital lobe, concerning for hemorrhage. Coronal brain MVI demonstrates very marked peri-gyral. The patient passed away while on ECMO, nine days after cannulation.

Lenticulostriate vessels in the BGT were evaluated in 30 patients. Vessel tortuosity was seen in 26/30 (86.7%) (Figure 1); blood flow was symmetric in 24/30 (80%) (Figure 2); there was blood flow heterogeneity in 21/30 (70%) (Figure 3); BGT vessels were engorged in 9/30 (30%) (Figure 4); and there was decreased blood perfusion in 12/30 (40%) of cases (Figure 2). All patients who passed away either during ECMO (n = 3) or within a year from ECMO cannulation (n = 7) demonstrated vessel tortuosity (Table 1). Regarding the cortical microvessels, we excluded six patients because the field of view of MVI did not include the cortex. Five patients (20.8%) showed white matter vascular engorgement (Figure 5), and ten patients (41.7%) had increased peri-gyral flow (Figure 6) (Table 2).

There were no statistically significant differences between the presence of MVI features in the BGT region and clinical outcomes or duration of ECMO (Table 1). On the other hand, in cortical evaluation, white matter vascular engorgement was significantly associated with any poor outcome, as defined by death, seizure, and/or cerebrovascular events on MRI (*p* = 0.03) (Table 2). There were no statistically significant differences between the presence of cortical MVI features when compared to the duration of ECMO.

IRR between pediatric radiologists ranged from none to strong depending on the feature evaluated. The MVI finding with the best IRR was white matter vascular engorgement in the cortex (0.89), followed by hypoperfusion (0.59) and engorgement (0.57) in the BGT microvessels. There was no agreement for the evaluation of symmetry and heterogeneity in the BGT region and for evaluating the presence of peri-gyral flow. IRR values for all MVI variables are presented in Table 3.

## 4. Discussion

We described for the first time the cerebral perfusion patterns using MVI in a cohort of infants undergoing ECMO and found that cortical white matter microvascular engorgement is associated with poor outcomes in this population. Our study demonstrates that cerebral MVI is feasible for evaluating critically ill patients and offers opportunities for noninvasive assessment of cerebral blood flow (CBF) in neonates and infants for outcomes prediction during ECMO.

The study shows feasibility of visualization and monitoring of whole brain microvascular flow real-time using MVI during ECMO. Grayscale US is the standard of care and allows detection of major cerebrovascular events such as hemorrhage [21,22]. MVI supplements conventional tools such as grayscale US by providing functional insights into the brain, namely helping to measure cerebral blood flow and its surrogate which are important in assessing tissue viability and risk of injury [23,24,25]. As compared to transcranial Doppler (TCD), which helps assess macrovascular flow dynamics, MVI helps assess microvascular flow dynamics which is an important advantage in ECMO patients at higher risk of acute cerebrovascular events related to the circuit, anticoagulation, and/or underlying condition [1]. Alternate modalities allowing assessment of cerebral microvascular perfusion include near infrared spectroscopy (NIRS) and diffuse correlation spectroscopy (DCS) but with these technologies, neither direct visualization of flow dynamics nor whole brain imaging can be achieved. There is a case report of contrast-enhanced US (CEUS) being used in ECMO; however, further work is needed to validate its safety and utility. Our study sets the stage for potential integration of MVI into brain US protocols for studying spatiotemporal evolution of brain perfusion during ECMO.

Interestingly, we found that cortical white matter microvascular engorgement is associated with poor outcomes during and post ECMO. This has significant clinical implications as the validation of our findings in a larger cohort can lead to developments in early intervention and therapeutic strategies. While the pathophysiology behind our observed finding warrants further investigation, this could be due to the severity of white matter injury, impaired autoregulation, and/or venous hypertension or congestion during ECMO [14,26,27]. Of note, increased peri-gyral flow may be due to profound hypoxia and subsequent loss of cerebral autoregulation, cerebral edema, and/or reperfusion injury [6]. While the basal ganglia and thalamic flow features were not significantly correlated with outcomes, high prevalence of lenticulostriate vessels was observed. Whether this is related to the circuit, autoregulation integrity, or microangiopathy in this cohort needs further exploration. In the future, there may be a potential to improve quantitative approach to assessing these flow features for personalized diagnostics and therapeutic guidance. Moreover, the combination of imaging biomarkers with serum biomarkers could potentially improve prognostication [28,29,30].

Our finding supplements the prior literature on the prognostic utility of TCD in the pediatric population, which has mixed results on the association between flow velocities and neurological outcomes [31,32,33]. Prior work by Rilinger and colleagues [33] demonstrated elevated middle cerebral artery and anterior cerebral artery velocities in 10-day-old or younger neonates. In older patients, systolic and diastolic velocities in the middle cerebral artery and internal carotid artery were conversely lower when compared with age-matched controls. After ECMO decannulation there were increased velocities regardless of patient’s age; however, there were no significant associations between velocities and outcomes in this cohort. On the other hand, O’Brien and colleagues suggested that patients who developed cerebral hemorrhage had higher systolic flow velocity, diastolic flow velocity, and mean flow velocity; nonetheless, this population included older children other than neonates and infants [32,33]. The advantage of MVI over TCD is the ability to visualize spatiotemporal heterogeneity in cerebral microvascular flows, such as the increased peri-gyral flow, which we find is of prognostic value. Furthermore, MVI helps to assess heterogeneous microvascular morphology which may be of importance in the pathophysiology of ECMO-induced brain injury. More importantly, focal and/or asymmetric injury patterns may be detected using MVI.

There have been recent advances in noninvasive quantification of cerebral blood volume flow at the bedside using US [34,35]. Busch and colleagues used DCS and frequency-domain diffuse optical spectroscopy (FD-DOS) to detect impaired cerebral autoregulation prior to brain injury instauration. Their results suggested that continuous optical monitoring was helpful in evaluating individuals longitudinally, as quantitative measurements varied considerably between patients [24].

In the future, multimodal imaging integrating MVI and optical technology may be an option to improve our functional insights into the brain during ECMO. Prior studies with NIRS have shown that the reduction in cerebral tissue oxygenation saturation (rScO2) is associated with poor outcomes [36,37,38]. This has been attributed to profound alterations in cerebral blood flow and autoregulation. Interestingly, reduced cerebral blood flow has been correlated with poor outcomes using this technology [36,37,38]. On the other hand, a rScO2 greater than 80% can be a protective factor for in-hospital mortality [39]. It is important to acknowledge that optical techniques measure blood flows in the superficial cortex such that flow alterations in the cortical white matter as measured in our study cannot be evaluated. Thus, the technique, while providing valuable metabolic insights, may benefit if integrated with MVI.

We acknowledge the limitations of this study. First, its retrospective nature carries an inherent risk of selection bias; moreover, we had a relatively small sample size as this was a feasibility study. Second, the scans were acquired by different sonographers; as US is an operator-dependent modality, there might be technical and operative differences that could have affected the quality of the studies and ultimately impaired the visualization of microvessels in the cortical and the BGT regions. Third, there are several clinical and laboratory variables beyond congenital heart disease that we did not consider and could act as confounders when predicting morbidity and mortality in our population. Fourth, there was a poor inter-rater agreement for certain brain MVI features; however, these discrepancies were resolved by consensus of the two radiologists.

In conclusion, MVI is a feasible modality to evaluate cerebral perfusion in infants undergoing ECMO. Additionally, evidence of white matter vascular engorgement after ECMO cannulation could serve as a predictor of poor outcomes in this population and can be reliably discerned by multiple readers. This technology is promising as it allows cerebral blood perfusion evaluation non-invasively and at the bedside, potentially reducing costs and logistic burden compared to other modalities. Although this initial experience is favorable, a prospective, longitudinal evaluation in a larger cohort is warranted to determine the role of MVI in ECMO while considering other clinical and paraclinical variables.

## Figures and Tables

**Table 1:** Evaluation of microvessels in the BGT region with microvascular imaging ultrasound.

BGT Vessels (n = 30)												
Vascular Features in MVI Exam	Clinical History		Outcomes									
	History of Congenital Cardiac Disease (n = 15)		Any Poor Outcome ^$^ (n = 17)		Death during ECMO (n = 3)		Death (n = 7)		Cerebrovascular Event (n = 7)		Seizures (n = 3)	
	n/15 (%)	*p*-Value	n/17 (%)	*p*-Value	n/3 (%)	*p*-Value	n/7 (%)	*p*-Value	n/7 (%)	*p*-Value	n/3 (%)	*p*-Value
Tortuosity (n = 26)	13/15 (86.6%)	1	16/17 (94.1%)	0.17	3/3 * (100%)	0.47	7/7 * (100%)	0.24	7/7 * (100%)	0.24	2/3 (66.6%)	0.28
Asymmetry (n = 6)	3/15 (20%)	1	5/17 (29.4%)	0.14	0/3 (0%)	0.36	4/7 (57.1%)	0.52	2/7 (28.6%)	0.52	1/3 (33.3%)	0.54
Heterogeneity (n = 21)	10/15 (66.6%)	0.69	12/17 (70.6%)	0.94	2/3 (66%)	0.89	5/7 (71.4%)	0.93	4/7 (57.1%)	0.39	3/3 * (100%)	0.23
Engorgement (n = 9)	4/15 (26.7%)	0.69	7/17 (41.2%)	0.13	1/3 (33.3%)	0.89	2/7 (28.6%)	0.93	3/7 (42.9%)	0.39	2/3 (66.6%)	0.14
Hypoperfusion (n = 12)	6/15 (40%)	1	7/17 (41.2%)	0.88	1/3 (33.3%)	0.80	3/7 (42.9%)	0.86	3/7 (42.9%)	0.86	1/3 (33.3%)	0.80

BGT: basal ganglia-thalami; US: ultrasound; MVI: microvascular imaging; ECMO: extracorporeal membrane oxygenation. ^$^ Included death, cerebrovascular events, and/or seizure within a year of ECMO cannulation. * Represents all patients with the condition in that column.

**Table 2:** Evaluation of cortical microvessels with microvascular imaging ultrasound.

Cortical Vessels (n = 24)												
Vascular Features in MVI Exam	Clinical History		Outcomes									
	History of Congenital Cardiac Disease (n = 12)		Any Poor Outcome ^$^ (n = 14)		Death during ECMO (n = 2)		Death (n = 6)		Cerebrovascular Event (n = 5)		Seizures (n = 3)	
	n/12 (%)	*p*-Value	n/14 (%)	*p*-Value	n/2 (%)	*p*-Value	n/6 (%)	*p*-Value	n/5 (%)	*p*-Value	n/3 (%)	*p*-Value
White matter vascular engorgement (n = 5)	3/12 (25%)	0.62	5/14 ^#^ (35.7%)	0.03~	1/2 (50%)	0.29	2/6 (33.3%)	0.38	2/5 (40%)	0.24	1/3 (33.3%)	0.57
Peri-gyral flow (n = 10)	7/12 (58.3%)	0.09	8/14 (57.1%)	0.07	2/2 * (100%)	0.08	3/6 (50%)	0.63	3/5 (60%)	0.35	2/3 (66.6%)	0.35

BGT: basal ganglia-thalami; US: ultrasound; MVI: microvascular imaging; ECMO: extracorporeal membrane oxygenation. ^#^ Statistically significant association. * Represents all patients with the condition in that column. ^$^ Included death, cerebrovascular events, and/or seizure within a year of ECMO cannulation.

**Table 3:** Inter-rater reliability for the detection of MVI findings.

Vascular Features in MVI Exam	Reviewer 1 vs. Reviewer 2	Reviewer 1 vs. Consensus	Reviewer 2 vs. Consensus
BGT vessels			
Tortuosity	0.53	0.35	0.63 *
Asymmetry	0.11	0.29	0.27
Heterogeneity	0.14	0.49	0.30
Engorgement	0.57	0.72 *	0.66 *
Hypoperfusion	0.59 *	0.66 *	0.66 *
Cortical vessels			
White matter vascular engorgement	0.89 *	0.88 *	0.78 *
Peri-gyral flow	0.03	0.73 *	0.07

MVI: microvascular imaging ultrasound; BGT: basal ganglia-thalami. * These interpretations showed moderate or strong level of agreement.

## Data Availability

Data are available from the corresponding author upon reasonable request.
